# Investigation of 12 X-STR loci in Mongolian and Eastern Han populations of China with comparison to other populations

**DOI:** 10.1038/s41598-018-22665-3

**Published:** 2018-03-09

**Authors:** Ruiyang Tao, Jiashuo Zhang, Yingnan Bian, Rixia Dong, Xiling Liu, Chao Jin, Ruxin Zhu, Suhua Zhang, Chengtao Li

**Affiliations:** 1 Institute of Forensic Medicine, West China School of Basic Medical Sciences & Forensic Medicine, Sichuan University, Chengdu, 610041 P.R. China; 2 Shanghai Key Laboratory of Forensic Medicine, Shanghai Forensic Service Platform, Academy of Forensic Sciences, Ministry of Justice, P.R. China, Shanghai, 200063 P.R. China; 3 Department of Forensic Science, Medical School of Soochow University, Suzhou, 215123 P.R. China; 4 The Affiliated Guangji Hospital of Soochow University, Suzhou, 215008 P.R. China; 5 Shanghai OE Biotechnology Co, Ltd, Shanghai, 201114 P.R. China

## Abstract

Due to the unique inheritance pattern, X-chromosomal short tandem repeats (X-STRs) have several advantages in complex kinship cases, such as deficiency cases or grandparent-grandchild and half-sisters testing. In our study, 541 unrelated individuals gathered from Mongolian and Eastern Chinese Han populations were successfully genotyped using the Investigator Argus X-12 kit. We calculated allele/haplotype frequencies and other forensic parameters of the two populations and further explored their genetic distance with already published Chinese populations and six global populations. Our results showed that the 12 X-STR markers were highly informative in the two populations when compared with nine other Chinese populations: significant differences were found at several loci. Geographically neighboring populations or different ethnic groups within the same area appeared to have closer evolutionary relationships. We also analyzed population genetic structure by performing clustering with the STRUCTURE program and Principal Coordinate Analysis (PCoA), and we found that the Chinese and other populations enrolled in this study could be distinguished. Furthermore, Mongolian males were distinguishable from the other studied males by a moderate genetic distance. Our study also expanded the X-STR database, which could facilitate the appropriate application of the 12 X-STR markers in the forensic field in China.

## Introduction

Short tandem repeats (STRs) are relatively mature genetic markers that were widely used in forensic applications. Genotyping of X-chromosomal STR (X-STR) markers has developed into a valuable method used by many forensic researchers as a supplement to the information provided by autosomal STR (AS-STR), Y-chromosomal STR (Y-STR), and mitochondrial DNA, particularly in deficiency and complex kinship cases^1,2^. These different approaches can complement each other with their characteristic genetic information. For instance, the ancestral information of populations deduced from genetic analyses could be quite different depending on the type of genetic marker used^3^. Meanwhile, in the identification of some certain kind of mixed stains, X-STRs can be used to exclude male individuals from the same paternal line. Moreover, because of the unique genetic inheritance of the X chromosome, the use of X-STRs is of great benefit in the identification of victims of war or mass disaster^4^.

There are several X-STR multiplexes being applied to forensic cases and genetics research. Among them, the Investigator Argus X-12 kit (Qiagen, Hilden, Germany), which includes 12 X-chromosomal STR markers as well as the amelogenin locus, has been used worldwide in a number of population sets^5–9^. To our knowledge, five Chinese Han subpopulations^10–14^ and eight ethnic minority groups from China^15–19^ have been previously studied using this kit. There are 56 ethnic groups in China, which has the largest population and third largest territory in the world. Each ethnic group has different geographical distributions, religions, customs, etc. Throughout history, some of these groups have been relatively isolated from each other, which may have resulted in relatively stable genetic differences between populations in defined areas. Therefore, for a better forensic application of the 12 X-STRs, it is important to understand the similarities or differences in genetic distance between different Chinese populations.

The purpose of this study was to investigate the frequency distribution data and forensic parameters of 12 X-STR loci in Mongolian and Eastern Chinese Han unrelated individuals as well as to illustrate the X-chromosomal evolutionary relationships between different Chinese populations and in comparison to other global populations. Additionally, this study enriched the X-STR database in order to highlight its significance in forensic identification and kinship analysis in different ethnic populations of China.

## Materials and Methods

### Population samples and DNA extraction

More than 70% of China’s Mongolian population lives in Inner Mongolia, which is located on the Eurasian continent^20^. Eastern China also has a large Mongolian population and accounts for 29.3% of the total population of China. In our study, samples from 541 healthy unrelated individuals (116 males and 116 females for the Mongolian samples, 200 males and 109 females for the Eastern Chinese Han samples) were collected. Blood samples were obtained from volunteer donors had information available under informed consent, following the protocols approved by the ethics committee at the Academy of Forensic Science, Ministry of Justice, P.R. China. DNA isolation was carried out by a Chelex-100 extraction protocol^21^. All the methods were carried out in accordance with the approved guidelines of the Academy of Forensic Sciences, Ministry of Justice, P.R. China.

### PCR amplification and capillary electrophoresis

All DNA samples and positive control samples (9947 A and XX28) were amplified with the Investigator Argus X-12 kit (Qiagen, Hilden, Germany) on a GeneAmp PCR System 9700 Thermal Cycler (Thermo Fisher Scientific, MA, USA), according to the manufacturer’s protocol. PCR products were detected by capillary electrophoresis on an ABI PRISM 3130*XL* Genetic Analyzer (Thermo Fisher Scientific, MA, USA) with the protocol guide for genotyping and the allele designation was analyzed using the Genemapper ID v3.2.1 software (Thermo Fisher Scientific, MA, USA).

### Statistical analysis and population comparisons

Allelic frequencies of the 12 X-STRs were calculated with CERVUS software/PowerStats v1.2.xls^22^ for female samples and were counted for male samples. Arlequin v3.5.2^23^ was used to calculate haplotype frequencies of the four linkage groups in male samples; Hardy-Weinberg equilibrium (HWE) in female samples; the exact test of population differentiation based on allelic frequencies of males and females; linkage disequilibrium (LD) in males and females; and pairwise F_st_ genetic distances per locus among Mongolian, Eastern Chinese Han and nine other published populations from China^10–14,16–19^. Forensic parameters for 12 X-STRs including polymorphism information content (PIC), homozygosity (HOM), heterozygosity (HET), power of exclusion (PE), paternity index (PI), paternity exclusion chance in duos and trios (MEC_D_ and MEC_T_)^24^, as well as power of discrimination for males (PD_M_) and females (PD_F_) were calculated with pooled allelic frequencies of the two populations using the online tool of the Forensic ChrX Research database (http://www.chrx-str.org).

China map was generated by and Package (ggplot2) and (maptools) of R: A Language and Environment for Statistical (https://www.R-project.org). The evolutionary history of eleven Chinese populations was inferred using the Unweighted Pair Group Method with Arithmetic mean (UPGMA) method^25^, and the optimal tree with the sum of branch length = 0.00821881 was shown (next to the branches). The evolutionary distances were used for phylogenetic analyses conducted in MEGA7 software^26^. Detailed population genetic structure analysis was performed with STRUCTURE v2.3.4^27,28^ under the condition of the admixture model and correlated allelic frequencies between populations. We analyzed the structure of Mongolian/Eastern Chinese Han populations and nine other populations from previous studies based on the same 12 X-STRs. The analysis results were uploaded to the Structure Harvester^29^ to detect the true K value of the data. Further comparisons among different male populations based on the Nei’s unbiased genetic distance^30,31^ were calculated and Principal Coordinate Analysis (PCoA, known previously as PCA)^32^ was performed using GENALEX 6.5 software.

### Data Availability

All data generated during this study are included in this published article (and its Supplementary Information files) and from the corresponding author on reasonable request.

## Results and Discussion

### HWE and LD test within populations

The results of HWE and LD tests in Eastern Chinese Han were presented in our previous report^33^. In the Mongolian samples, a total of 164 alleles were typed using the Argus X-12 kit and no statistically significant deviation from HWE was observed except for the DXS10134 locus in female samples (p = 0.0027) even after applying the Bonferroni’s correction for multiple testing (p = 0.05/12). This deviation from HWE may be caused by the substructure of the chosen population, which could result in a bias of the samples. In addition, no statistically significant LD was found in Mongolian females after Bonferroni’s correction (p = 0.05/66), which was different from the Eastern Chinese Han analysis. This might be due to the rigorous statistical methods. On the other hand, statistically significant LD was found between DXS10101 and DXS10103 loci in Mongolian males (p < 0.0001), which has also been reported in other populations^5,34,35^.

### Haplotype analysis in males

The 12 X-STR markers are clustered into 4 linkage groups based on their physical localizations: LG1 (Xp22) DXS8378-DXS10135-DXS10148, LG2 (Xq11) DXS7132-DXS10074-DXS10079, LG3 (Xq26) DXS10101-DXS10103-HPRTB, LG4 (Xq28) DXS7423-DXS10134-DXS10146, and thus each cluster of 3 markers is considered as one haplotype for the genotyping of males. The numbers of observed haplotypes in Mongolian males were 99, 71, 76 and 81 for LG1, LG2, LG3 and LG4, respectively (Supplementary Table S1); while in Eastern Chinese Han males the numbers were 159, 94, 101 and 114 for LG1, LG2, LG3 and LG4, respectively (Supplementary Table S2). Haplotype diversity (HD) of the four LGs varied from 0.9901 (in LG2) to 0.9972 (in LG1) in Mongolian males and varied from 0.9876 (in LG3) to 0.9973 (in LG1) in Eastern Chinese Han males. This illustrated that LG1 was the most polymorphic group with a frequency of 0.0259 for the most common haplotype in Mongolian males, 10-21-24.1 and 10-21-25.1 (for DXS8378-DXS10135-DXS10148), and a frequency of 0.0200 for the most common haplotype in Eastern Chinese Han males, 10-25-27.1. Haplotype 12-16-32 (for HPRTB-DXS10103-DXS10101) was the most common, observed 12 times in LG3 of the Eastern Chinese Han samples with a population frequency of 0.0600. The results showed that the four closely linked X-STR groups are highly informative genetic markers in the two studied populations.

### Genetic variability

The allelic frequency and forensic parameters of Eastern Chinese Han were also presented in our previous report^33^. Based on the calculated allelic frequencies of 12 X-STRs of Mongolian females and males, no significant difference was found between them (p > 0.05) by the Exact Test; therefore, males and females were pooled for calculating forensic parameters. The allelic frequencies of 12 X-STRs are shown in Supplementary Table S3. The results indicated that all 12 X-STR markers were polymorphic. The DXS10135 locus showed the highest forensic efficiency in both Mongolian (PIC = 0.9070) and Eastern Chinese Han populations, while DXS7423 was the least informative in both Mongolian (PIC = 0.4597) and Eastern Chinese Han populations. HET values varied from 0.5268 (DXS7423) to 0.9134 (DXS10135) in the Mongolian population. All the forensic parameters of 12 X-STRs for the Mongolian population are shown in Table 1. In total, the combined PD_F_, PD_M_, MEC_D_ and MEC_T_ were calculated as exceeding 0.9999999999, 0.9999999983, 0.9999987070 and 0.9999999931, respectively. All these forensic parameters demonstrated that the 12 X-STR markers were highly polymorphic and will be useful in forensic applications or anthropological research among the Mongolian population of Inner Mongolia and the Eastern Chinese Han population.

**Table 1:** Forensic parameters calculated with pooled allele frequencies of the Mongolian population.

Locus	PIC	HOM	HET	PE	PI	PD_F_	PD_M_	MECK_rüger_	MECK_ishida_	MEC_Desmarais_	MEC_Desmarais Duo_
DXS8378	0.5558	0.3896	0.6104	0.3036	0.1948	0.7936	0.6104	0.3608	0.5558	0.5558	0.4079
DXS10135	0.9070	0.0866	0.9134	0.8228	0.0433	0.9862	0.9134	0.8256	0.9070	0.9070	0.8363
DXS10148	0.9043	0.0889	0.9111	0.8181	0.0445	0.9853	0.9111	0.8201	0.9041	0.9043	0.8317
DXS7132	0.6975	0.2590	0.7410	0.4945	0.1295	0.8895	0.7410	0.5081	0.6975	0.6975	0.5583
DXS10074	0.7444	0.2213	0.7787	0.5600	0.1107	0.9167	0.7787	0.5693	0.7442	0.7444	0.6130
DXS10079	0.8032	0.1738	0.8262	0.6486	0.0869	0.9467	0.8262	0.6538	0.8032	0.8032	0.6867
DXS10101	0.8889	0.1026	0.8974	0.7901	0.0513	0.9810	0.8974	0.7944	0.8889	0.8889	0.8083
DXS10103	0.7496	0.2180	0.7820	0.5660	0.1090	0.9201	0.7820	0.5779	0.7496	0.7496	0.6194
HPRTB	0.6594	0.2926	0.7074	0.4398	0.1463	0.8663	0.7074	0.4643	0.6594	0.6594	0.5158
DXS7423	0.4597	0.4732	0.5268	0.2121	0.2366	0.7090	0.5268	0.2728	0.4596	0.4597	0.3181
DXS10134	0.8323	0.1505	0.8495	0.6939	0.0753	0.9602	0.8495	0.7017	0.8323	0.8323	0.7268
DXS10146	0.8670	0.1209	0.8791	0.7530	0.0605	0.9733	0.8791	0.7561	0.8670	0.8670	0.7753

PIC: polymorphism information content; HOM; homozygosity; HET: heterozygosity; PE: power of exclusion; PI: paternity index; PD_M_: power of discrimination in males; PD_F_: power of discrimination in females; MECK_rüger_: mean exclusion chance according to Krüger *et al*.; MEC_Kishida_: mean exclusion chance according to Kishida *et al*. MEC_Desmarais_: mean exclusion chance in standard trios involving daughters according to Desmarais *et al*.; MECDe_smarais Duo_: mean exclusion chance in father/daughter or mother/son duos according to Desmarais *et al*.

### Inter-population comparison

**Figure 1:**
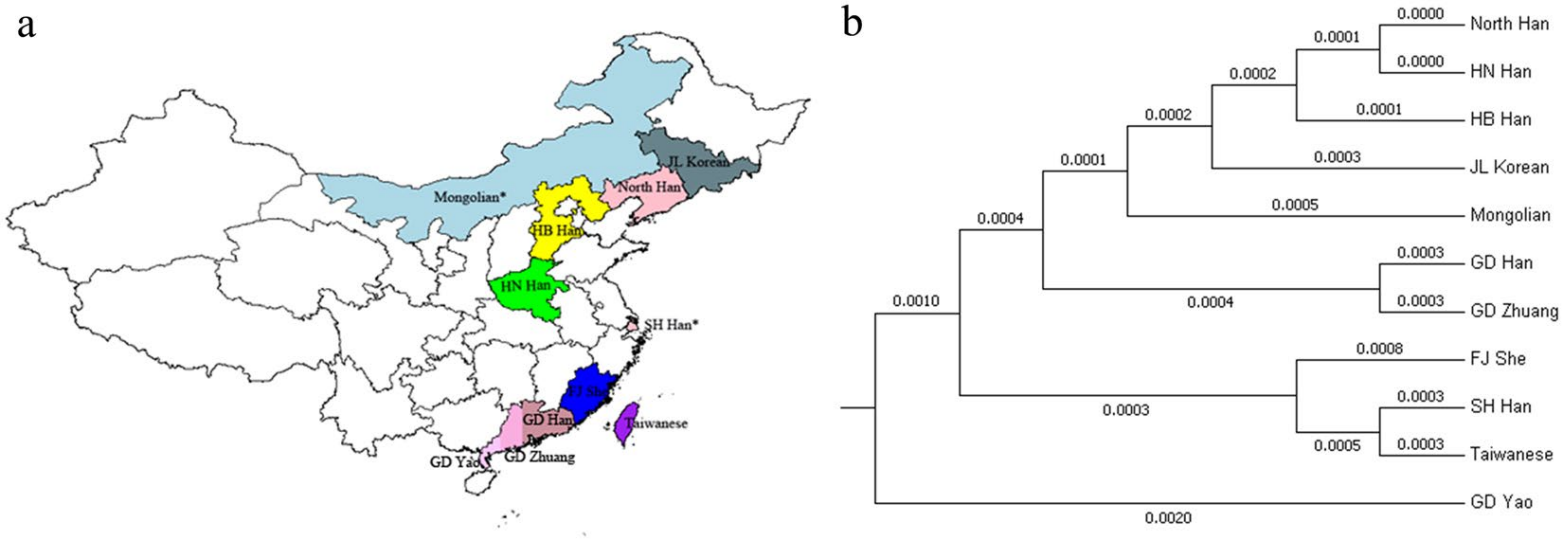
(**a**) Geographical distribution of 11 populations on the map of China, which was generated by Package (ggplot2) and (maptools) of R: A Language and Environment for Statistical (https://www.R-project.org). The areas marked by asterisks show the populations in our study. (**b**) Evolutionary relationships of 11 populations in different areas of China shown by a phylogenetic tree that was constructed based on 12 X-STRs.

For a better understanding of the genetic structures of these 12 X-STRs in Chinese populations, we assembled data from five Chinese Han subpopulations and four ethnic minority groups from different areas of China (Fig. 1a, Supplementary Table S4). Detailed genotyping data and allelic frequencies of these populations assayed by Investigator Argus X-12 kit were available in previously published reports. By calculation and comparison, we noticed that all the combined PD_F_ of the 11 populations were greater than 0.999999999, except for the Guangdong Yao ethnic group (cPD_F_ = 0.999999998); the combined PD_M_ ranged from 0.999999997 to 0.999999999; and the combined MEC_D_ and MEC_T_ ranged from 0.999998065 to 0.999999993 and 0.999998732 to 0.999999998, respectively. The abovementioned forensic parameters perfectly illustrated that the 12 X-STRs had great application value in forensic identification and kinship analysis among Chinese populations.

Next, the allelic frequencies of Mongolian and Eastern Chinese Han populations were compared with nine other Chinese populations. Since the F_st_ value is usually used for analyzing genetic distances between populations^36,37^, we calculated the pairwise F_st_ genetic distances (shown in Supplementary Table S5) among the 11 Chinese populations to study their phylogenetic relationships with each other. Based on these data, inter-population comparisons were performed: Significant differences were observed between Mongolian and Taiwanese populations at 4 loci; Mongolian and Guangdong Yao populations at 3 loci; Mongolian and Eastern Chinese Han populations at 2 loci; and for 1 locus between Mongolian and Han populations of Liaoning and Henan, Mongolian and Liaoning Korean populations, and Mongolian and Guangdong Zhuang populations. No significant difference was observed between Mongolian and Hebei Han, Guangdong Han, or Fujian She populations. The maximum F_st_ value was 0.0055 for the Guangdong Yao ethnic group and Taiwanese population.

### Phylogenetic tree by UPGMA phylograms

Despite the limited number of loci that can provide strong information about the general level of genetic diversity, the phylogenetic analyses indicated that populations that are geographically close tend to have shorter genetic distances compared to those that are geographically far apart, which is in accordance with the conventional knowledge of population genetics^38^. The visualized results of the observed genetic distances revealed that the Mongolian population in this study was genetically close to the populations from northern China, including the northern Han population and Korean ethnic population in the Liaoning province. On the other hand, the eastern/southern populations of China were not as related to the Mongolian population based on X-STRs analyses, regardless of ethnic identity. Of all the populations presented, the Guangdong Yao ethnic group was relatively far from the other populations according to the phylogenetic tree we constructed (shown in Fig. 1b). These observed results were consistent with the cultural and geographical background and development of the abovementioned populations.

### Clustering by STRUCTURE analysis

**Figure 2:**
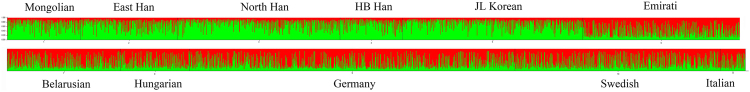
Estimated population genetic structure of eleven different populations at K = 2.

To further investigate the population genetic structure at an individual level on a worldwide scale, the STRUCTURE program was used to process the genetic data of five representative Chinese populations (Mongolian, Han from Eastern China, Han from Liaoning province, Han from Hebei province, and Korean from Jilin province)^10,11,16,33^, as well as six extra published populations from European and Asia^39–44^. The range of possible Ks was tested from 2 to 11. According to the Evanno method^45^, when the real K is indeterminate under the interference of Ks plateaus in the distribution of L (K), it often locates at the modal value of the distribution of ΔK; therefore, the most appropriate K was 2 (Supplementary Figure S1). The clustering of the populations presented an easily distinguishable geographic pattern (Fig. 2). Mongolian, Eastern Chinese Han and the other Chinese populations belonged to cluster 1, while the other six global populations belonged to cluster 2. When K = 3–11, there were no recognizable boundaries within the Chinese populations by STRUCTURE analysis. The same situation was also observed in cluster 2 (the Emirati, Belarusian, Hungarian, Germany, Swedish and Italian populations): the five geographically close Western European populations and the Emirati from United Arab Emirates (UAE), who live in the tri-continental crossroads connecting Africa, Europe and Asia, shared a relatively consistent genetic structure pattern no matter how the K value was assumed (Supplementary Figure S2). These results conformed to the findings of Brissenden *et al*. based on an autosomal SNP study^46^ and indicated that the Mongolian and Eastern Chinese Han were inseparable from other Chinese at the genetic level from STRUCTURE analysis.

### Principal Coordinate Analysis (PCoA)

**Figure 3:**
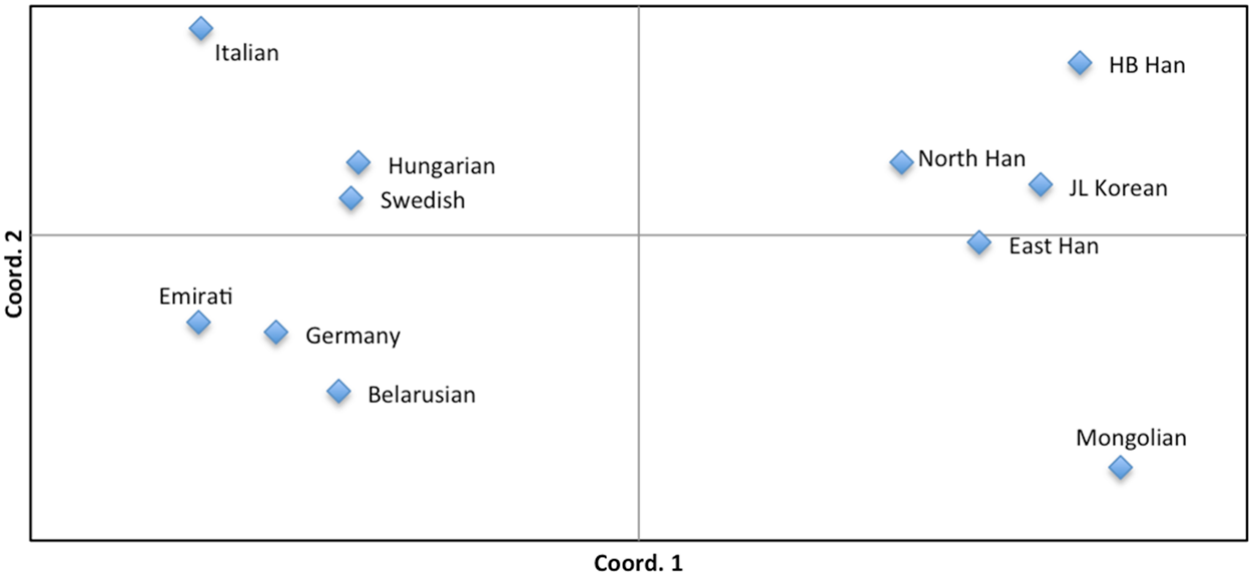
Principal Coordinate Analysis (PCoA) plot displaying the genetic relationships among five Chinese male populations and six male populations from Europe and Asia based on Nei’s unbiased genetic distance matrix of 12 X-STRs.

The genetic pattern of the male X chromosome inherited from the mother, normally referred to as the haplotype data set, makes the genetic distribution of X-STRs in males worth exploring. PCoA provides a way of visualizing the essential patterns of genetic relationships contained in a special matrix (e.g., distance matrix) and allows us to find and plot the major patterns within a multivariate data set. PCoA gives a reasonable assumption at a higher taxonomic level, compared to the algorithms that always assume a hierarchical genetic structure in Tree building methods. In this respect, PCoA is an effective complement to the evolutionary tree. Therefore, PCoA was carried out to test for significant variation in the genetic distribution of 12 X-STR markers among the males of abovementioned eleven populations in the STRUCTURE analyses. As shown in Fig. 3, the first two principal components explained 94.62% of the total variance observed within these populations (the first and second component accounted for 88.02% and 6.60%, respectively). In the PCoA diagram, Mongolian, Eastern Chinese Han and other Chinese populations were clustered together on the right, while other global populations were clustered on the left. Among the European male populations, Hungarian, Swedish and Italian were located in the upper left quadrant, while Germany and Belarusian were clustered together in the lower left quadrant, which was in accordance with the national stratification within Europe in other reports^41,43^. Meanwhile, the Emirati males from the Arabian Peninsula of Western Asia also located in the lower left quadrant and could not be distinguished from the Europeans by the 12 X-STRs, which was consistent with the result of the STRUCTURE analysis in our study. This may due to the complex biogeography and history background of UAE. Among the cluster of Chinese populations, Hebei Han, Liaoning Han and Korean from Jilin shared a closer genetic distance, which supported the results observed from the phylogenetic tree. Additionally, the Mongolian population was located in the lower right corner, slightly far from other Chinese groups in terms of genetic relationship. Similar results were found in a study of Mongolian Y-STRs, and this probably reflects customs and territory restriction to some extent^47^. Our analysis indicated that the 12 X-STR loci had some advantages in distinguishing Mongolian males from others by the PCoA method.

The Mongolian is a traditional nomadic nationality mainly distributed in Eastern Asia, it is one of the largest minority nationalities in China, and also the main nation of Mongolia. In addition, the Mongolians are also distributed in Asian and European countries such as Russia. The Mongolian nationality first originated from the eastern bank of the ancient Wangjian River. In the early thirteenth century, the Mongolian ministry, headed by Genghis Khan, unified the Mongolia region, and gradually formed a new national community. In the history of China and even in the world, the Mongolian people have had a profound influence and significant role in the economy, politics and culture. Besides, Eastern China is a densely populated area, the population there accounts for 29.3% of the total population of China, which has the most common characteristics of Chinese. Therefore, they are both very important and particular population groups in China, that’s why our study focused on them. Its particular genetic inheritance that makes the X chromosome valuable for human evolutionary and historical research^48,49^. On the one hand, there is a single copy of the X chromosome in males, it is possible to detect their haplotypes which are needed to infer the phylogeny of a region. The X/Y chromosome and mitochondrion are candidates for researches that use phylogenies, an advantage that explicates their dominant role in human historical studies^50–52^. On the other hand, recombination on women X chromosome also creates vast quantities of information that is vital for providing a complete view of the history of human populations. Although the bulk of human historical researches have been done with Y chromosome or mitochondrion, X chromosome has the advantage of existing in both genders and much lower rates of genetic drift^50^. The X-STR markers included in Argus X-12 kit are the most developed and widely used, there have been a great amount of 12 X-STRs data of different populations, by which we can conduct the analysis of population characteristics or geographical studies. Therefore, we conducted genetic analysis of Mongolian and Eastern Chinese Han populations based on 12 X-STR markers and achieved significant and prominent results that might be used in more precise substructure detection of Eastern Asian population and the estimation of individual biogeographical ancestry in further study.

## Conclusions

Our current study indicated that the Investigator Argus X-12 kit used in Mongolian and Eastern Chinese Han sample sets provided highly polymorphic data for discriminating individuals and testing kinship, which enriched the Chinese ethnic genetic information. The significant deviations from HWE observed at the DXS10134 locus in the Mongolian population could possibly be eliminated by increasing the sample size from geographically closed regions in Inner Mongolia. The genetic comparison among eleven Chinese populations demonstrated that the statistically significant genetic distances were consistent with the known situation of these national mixed, small-settled and staggered populations living in China. In addition, neighboring populations and different ethnic groups within the same area appeared to have closer evolutionary relationships in the phylogenetic tree. The STRUCTURE analyses revealed that the Mongolian and Eastern Chinese Han populations belonged to the same cluster as the other Chinese populations, but were apparently distinct from other global populations. The PCoA analysis indicated that the 12 X-STRs could not only be applied in distinguishing Chinese males and the males from the abovementioned countries, but also able to show a moderate genetic distance between Mongolian and the other Chinese male populations. To our best knowledge, this is the first report to demonstrate that the 12 X-STR markers could be useful in dealing with the genetic relationship between Mongolian males and others.

However, for some populations, such as the Manchu and Miao with their large populations, or the Kazak with significant differences from other Chinese populations in other genetic markers, there are still no published data concerning the 12 X-STRs panel. Therefore, further studies should be focus on increasing the sample size of current ethnic groups and including more nationalities. Hopefully, our endeavor will help to establish an appropriate and integrated X-STR database that is suitable for the large population of China.

## Electronic supplementary material

Supplementary information

Table S1

Table S2

Table S3

Table S4

Table S5
