# Supplementary material for: Investigation of 12 X-STR loci in Mongolian and Eastern Han populations of China with comparison to other populations
Source: Sci Rep. 2018 Mar 9;8:4287. doi: 10.1038/s41598-018-22665-3 (PMC5844901; doi:10.1038/s41598-018-22665-3)
Supplement: Supplementary file 1 — Supplementary information [file 41598_2018_22665_MOESM1_ESM.pdf]

Investigation of 12 X-STR loci in Mongolian and Eastern Han populations of China with comparison to other populations

Ruiyang Tao[1,2], Jiashuo Zhang[3], Yingnan Bian[2], Rixia Dong[4], Xiling Liu[2],Chao Jin[5], Ruxin Zhu[2], Suhua Zhang[2]*, Chengtao Li[1,2]*

[1] Institute of Forensic Medicine, West China School of Basic Medical Sciences & Forensic Medicine, Chengdu 610041, China.

[2] Shanghai Key Laboratory of Forensic Medicine, Shanghai Forensic Service Platform, Academy of Forensic Sciences, Ministry of Justice, P.R. China, Shanghai 200063, P.R. China

[3] Department of Forensic Science, Medical School of Soochow University, Suzhou 215123, China.

[4] The Affiliated Guangji Hospital of Soochow University, Suzhou 215008, P.R. China

[5] Shanghai OE Biotechnology Co, Ltd, Shanghai 201114, P.R. China

* Corresponding author: lichengtaohla@163.com; zhangsh@ssfjd.cn.

Supplementary information:

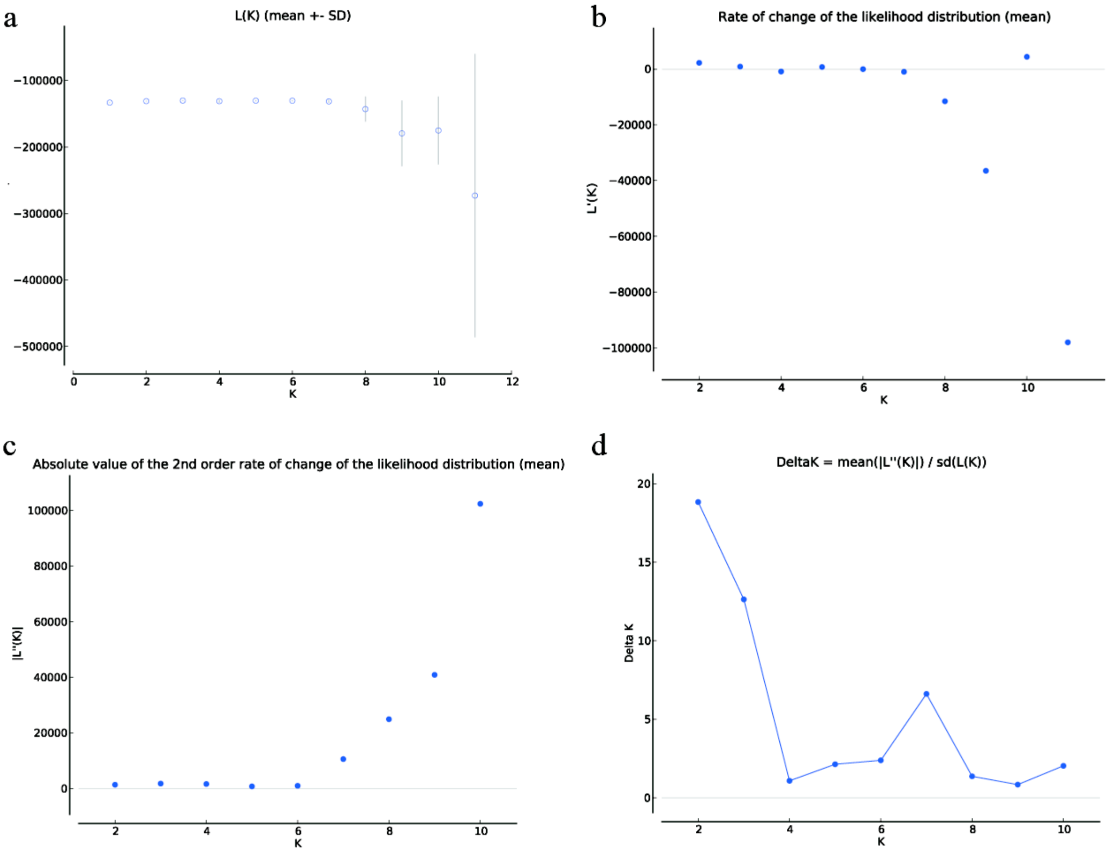

Supplementary Figure S1. Graphical method used to detect the true K value. (a) Mean L(K) (± SD) over 10 runs for each K value. (b) Rate of change of the likelihood distribution (mean ± SD). (c) Absolute values of the second order rate of change of the likelihood distribution (mean ± SD). (d) ΔK calculated as ΔK=m|L" (K)|/s[L(K)]. The modal value of this distribution is the true K (*) or the uppermost level of structure, here 2 clusters.

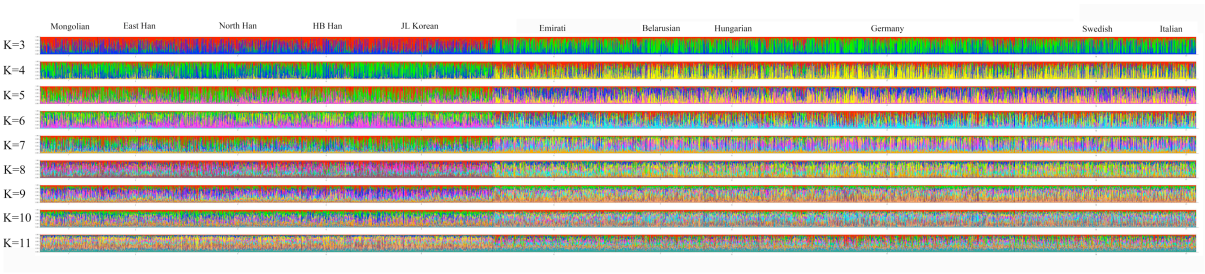

Supplementary Figure S2. Estimated population genetic structure of Mongolian, Eastern Chinese Han and the nine other populations when K=3-11.

Supplementary Table S1. Haplotype frequency in Mongolian males (N=116).

Supplementary Table S2. Haplotype frequency in Eastern Chinese Han males (N=200).

Supplementary Table S3. Allelic frequency in the Mongolian population (N=232, female=116, male=116).

Supplementary Table S4. A total of 11 Chinese population groups and their sample sizes for 12 X-STR population comparisons.

Supplementary Table S5. Average F_{ST} based on allele frequencies among 11 Chinese populations.
